# Symptoms trend and challenges in dental practice during delta variance COVID-19 pandemic in Indonesia: Google Trends Analysis

**DOI:** 10.12688/f1000research.134366.1

**Published:** 2023-07-10

**Authors:** Faizul Hasan, Noor Rohmah Mayasari, Eisner Salamanca, Odontuya Dorj, Rahmat Dani Satria, Kamaluddin Latief, Mokh. Sujarwadi, Hendrik Setia Budi

**Affiliations:** 1 School of Nursing, College of Nursing, Taipei Medical University, Taipei, Taiwan; 2 Department of Nutrition, Faculty of Health Sciences, Universitas Negeri Surabaya, Surabaya, Indonesia; 3 School of Dentistry, College of Dentistry, Taipei Medical University, Taipei, Taiwan; 4 Department Dental Technology and Dental Hygiene, School of Dentistry, Mongolian National University of Medical Sciences, Ulaanbaatar, Mongolia; 5 Department of Clinical Pathology and Laboratory Medicine, Faculty of Medicine, Public Health and Nursing, Universitas Gadjah Mada, Yogyakarta, Indonesia; 6 Global Health and Health Security Department, College of Public Health, Taipei Medical University, Taipei City, Taiwan; 7 Centre for Family Welfare, Faculty of Public Health, Universitas Indonesia, Depok, Indonesia; 8 Faculty of Nursing, Universitas Jember, Jember, Indonesia; 9 Department of Oral Biology, Faculty of Dental Medicine, Universitas Airlangga, Surabaya, Indonesia

**Keywords:** COVID-19, google trends, Dental care, symptoms

## Abstract

**Background:** The COVID-19 pandemic has grown to be a serious issue on a global scale. Dental care is one of the industries affected by COVID-19. The surveillance utilizing lifetime data, however, is still not clear. The purpose of this study was to use Google Trends (GT) analysis to examine symptom trends and challenges during the COVID-19 outbreak in Indonesia.

**Methods:** Covid-19 cases retrieve from Our World in Data. The cases were collected between 1 April 2021-30 September 2021. The GT was used to discover Indonesian relative search volume (RSVs) covering the timeframe of the first outbreak covid-19 pandemic in Indonesia on 1 March 2020 until 13 February 2022. The duration of the search was chosen to reflect the relative popularity of the keywords "symptoms and dentistry practice challenge-related terms" and "coronavirus". **Results:** We observed that there was a significant and positive correlation between the COVID-19 daily case using GT RSV data and the COVID-19 case from Our World in Data. The COVID-19 daily case had a strong correlation with search terms related to symptoms (such as fever, sore throat, flu, toothache, and cough), drugs (such as ibuprofen, paracetamol, demacolin, bodrex, and antibiotic), and health management (such as self-isolation and telemedicine).

**Conclusion:** Using GT may be helpful to observe the current symptoms trends as well as its challenge tendencies as a surveillance tool for a continuing pandemic like COVID-19. GT should be considered and used as it has the potential to be a powerful digital epidemiology tool that can provide more insight into disease dynamics.

## Introduction

The coronavirus disease 2019 (COVID-19) was firstly found in China, ^1^ was a virus that have been infected many people and caused many deaths worldwide. ^2^ Unlike a severe acute respiratory syndrome (SARS) ^3^^–^^5^ the COVID-19 incubation period was longer (4–12 days) than that of SARS (2–7 days). ^6^ In addition, this virus, caused pandemic, has a rapid transmission speed and need special treatment approach. ^7^ People suffering from COVID-19 can developed several symptoms such as fever, headache, and dry cough. ^8^^,^^9^ Finding the best symptom management are clinically important.

In the early phases of the COVID-19 pandemic, dental healthcare professionals - including dentists, dental assistants, dental hygienists, and nurse practitioners - were aware of the significant risk of exposure. ^10^^,^^11^ It is documented that dentists have a significant risk of transmitting COVID-19 from their patients because of the transmission through respiratory droplets, the use of dental handpieces that produces aerosols, and their near physical closeness with patients. ^11^^–^^13^ Many dental clinics had not identified the spread of SARS-CoV-2 as a significant threat to their patients or themselves. The most recent research demonstrated that SARS-CoV-2 is not only present in saliva but also in the salivary glands, due to the salivary glands and tongue epithelium’s angiotensin-converting enzyme 2 ( *ACE2*) high expression. ^14^

The Centers for Disease Control and Prevention (CDC) advises people to cover their faces when in dental facilities and to take them off only when receiving treatment. It is nonetheless recommended that patients keep their distance from one another in order to reduce the risk of the virus spreading among potentially asymptomatic individuals. ^15^ By integrating information technology, telemedicine or telehealth provides online medical care to patients who are dispersed out across different locations. ^16^ Dental professionals can reduce patient interaction before acting by using teledentistry. ^17^ The dentist who is concerned about patient who have COVID-19, can find employing remote evaluation helpful. Subdivisions of teledentistry with significant roles in dental practice include teleconsultation, tele-diagnosis, teletriage, and telemonitoring. ^18^

Internet search has become the major source of information, including medical and dental terms. In recent years, the internet has established itself as a major resource of information. ^19^ Through keyword-driven internet searches, people have quick access to a vast amount of information. ^19^^,^^20^ It was estimated that about eighty percent of internet users have looked for health information through online platforms. ^21^ Big data such as Google Trends (GT) has become the largest potential major source of data for medical and dental studies that need to be properly analyzed and interpreted. ^22^ The GT service assesses the popularity of internet search queries and can be used as a monitoring tool in a variety of languages and locations around the world. ^23^ However, no study has investigated the search for symptoms trends and challenges in dental practice particularly during COVID-19 pandemic in Indonesian populations.

Providing life data trend using GT may provide important and updated information for clinical practitioners and health policy makers. The aim of this study was to analyze symptoms trend and challenges in dental practice during the COVID-19 pandemic in Indonesia using the Google trends analysis.

## Methods

### Confirmed cases of COVID-19 data

COVID-19 cases retrieve from https://github.com/owid/covid-19-data/tree/master/public/data/ maintained by Our World in Data. The cases collected between 1 April 2021-30 September 2021, this time periods were the occurrence of delta variance in Indonesia.

### Google trends

Google trends is available at https://trends.google.com.tw/trends/?geo=TW. Google trends was used to discover Indonesian relative search volume (RSVs) covering timeframe of the first outbreak COVID-19 pandemic in Indonesia on 1 March 2020 until 13 February 2022. The search period was used to reflect the relative interest in “coronavirus” and “symptoms and dental practice challenge-related keywords”. RSVs ranges from 0-100, with 100 being the highest relative search term activity for specifies search keyword in the time frame period of interest. Google trends only allows a maximum of five terms search, which is enabled to be compared.

### Search terms

Bahasa was used to identified search term. Prior to identifying included search terms in each category, we search the potential keyword to understand the Indonesian search interest related symptoms and dental practice challenge. We identified search terms in to three categories: (1) symptoms (e.g., demam, sakit tenggorokan, flu, sakit gigi, batuk); (2) drugs (e.g., ibuprofen, paracetamol, ponstan, demacolin, bodrex, antibiotik), health management (e.g., isolasi mandiri, isoman, telemedicine, teledentistry).

### Statistical analysis

To perform all statistical analyses, we used SPSS software version 23.0 (IBM, Armonk, NY, USA). Descriptive analysis using RSVs related keyword was used to identify the trend of symptoms and dental practice challenge during COVID-19 pandemic in Indonesia. A Kolmogorov-Smirnov test was used to test the normality data. The relationship between RSVs and coronavirus (1 April 2021-30 September 2021) was tested using Spearman’s rank-order correlation coefficient. The timeframe was chosen to emphasize the highest peak of interest search term during covid-19 pandemic in Indonesia. The correlation was interpreted use following category: r = 0.1-0.2 is very weak, r = 0.3-0.5 is fair, r = 0.6-0.7 is moderate, r = 0.8-0.9 is very strong and r = 1 is perfect. ^24^

## Results

### COVID-19 cases

**Figure 1.**
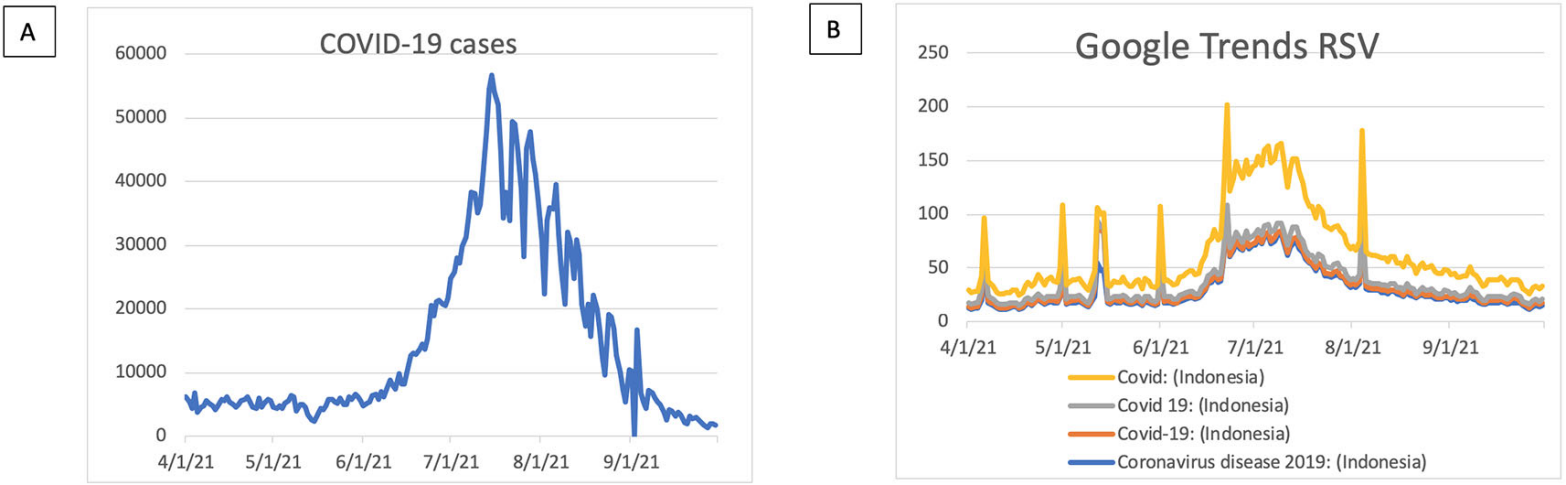
Covid-19 case trends. (A) COVID-19 case based on our Worlds Data. (B) COVID-19 based on Google Trends RSV. RSV = relative search volumes.

The trend of the COVID-19 case based on “Our Worlds Data” was seen in Figure 1A. The pick of COVID-19 cases sharply increased during June 2021 and was seen to have a downtrend after August 2021. In terms of COVID-19 cases using GT RSVs, we used four keywords of “Covid”, “Covid 19”, “Covid-19”, and “Coronavirus disease 2019” used to search the coronavirus-related terms ( Figure 1B). We found a similar trend in that the COVID-19 case increased after June 2021 and decreased after August 2021.

### Symptoms

**Figure 2.**
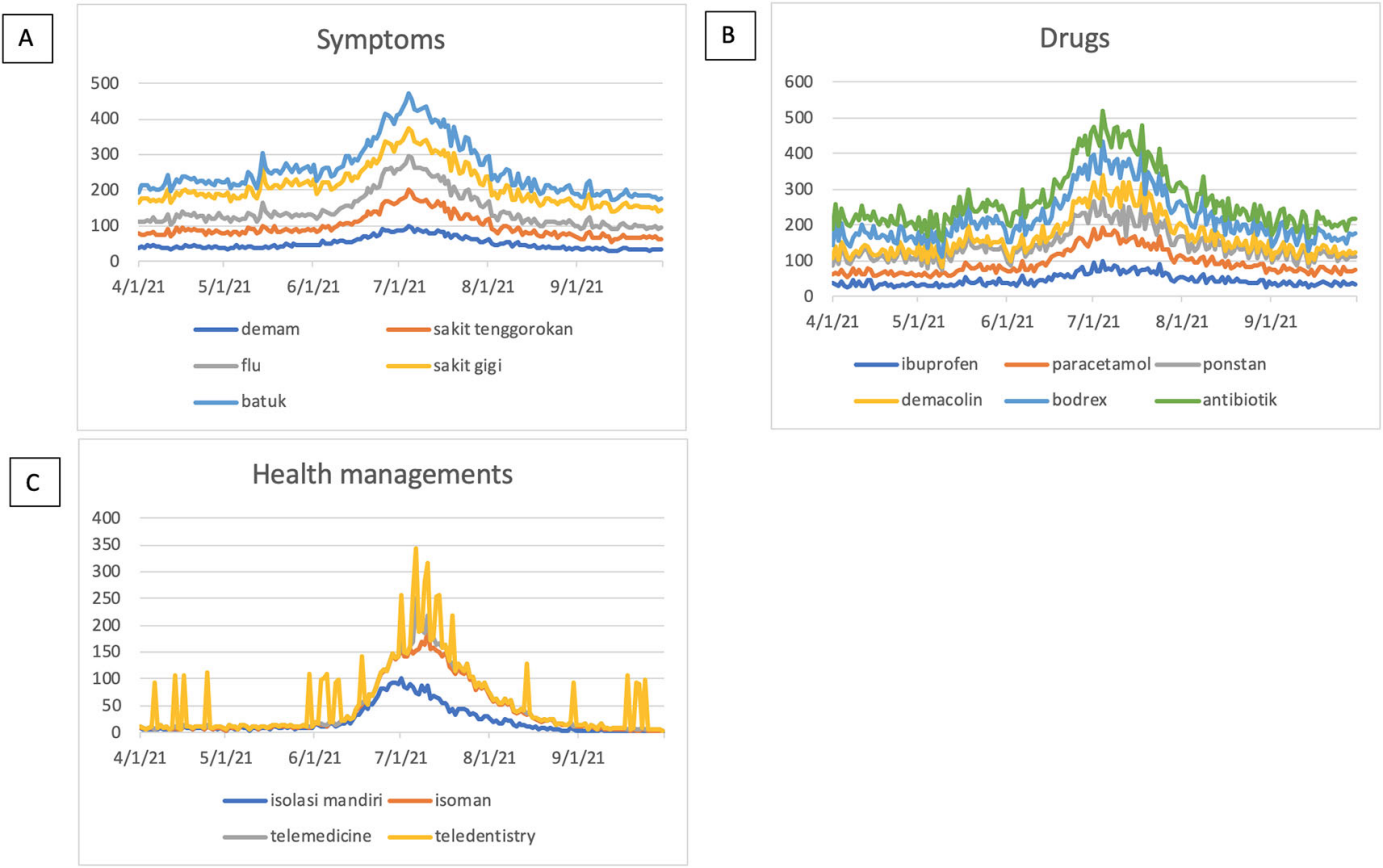
Google trend RSV curves-related search terms. (A) Symptoms-related search terms; fever (In: demam), sore throat (In: sakit tenggorokan), flu (In: flu), toothache (In: sakit gigi), cough (In: batuk). (B) Drugs-related search terms; ibuprofen, paracetamol, ponstan, demacolin, bodrex, antibiotic (In: antibiotik). (C) Health managements-related search terms; self-isolation (In; isolasi mandiri or isoman), telemedicine, teledentistry. RSV = relative search volumes.

**Figure 3.**
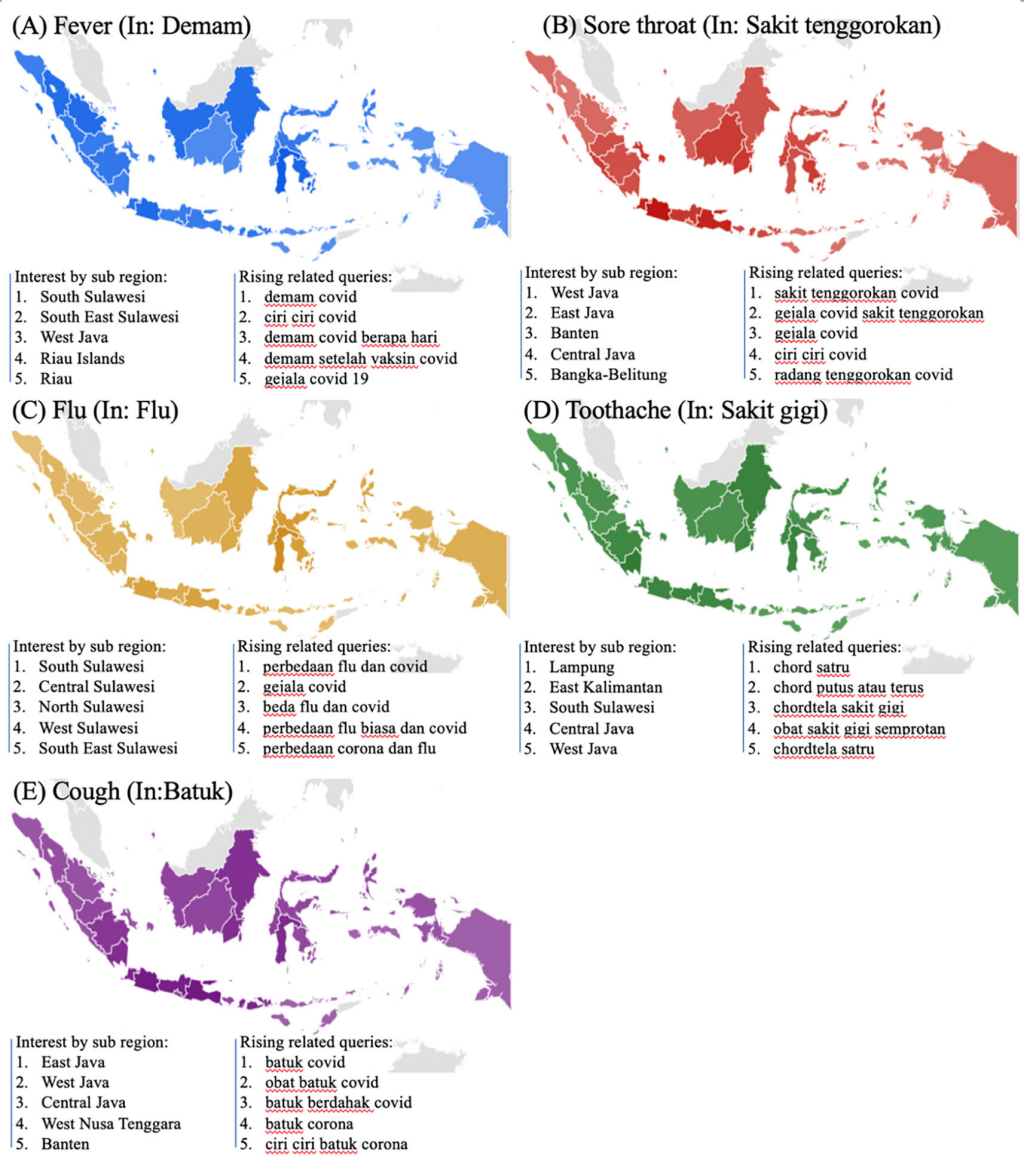
Google trend of symptoms RSV curves-related search terms.

The symptoms-related search terms including fever (In: demam), sore throat (In: sakit tenggorokan), flu (In: flu), toothache (In: sakit gigi), and cough (In: batuk) were seen in Figure 2A. The RSVs curves showed increased trends during the COVID-19 uptrend period between June, 2021 and August, 2021. The top five interest by sub region of those symptoms mentioned above were depicted in Figure 3A-E.

### Drugs

**Figure 4.**
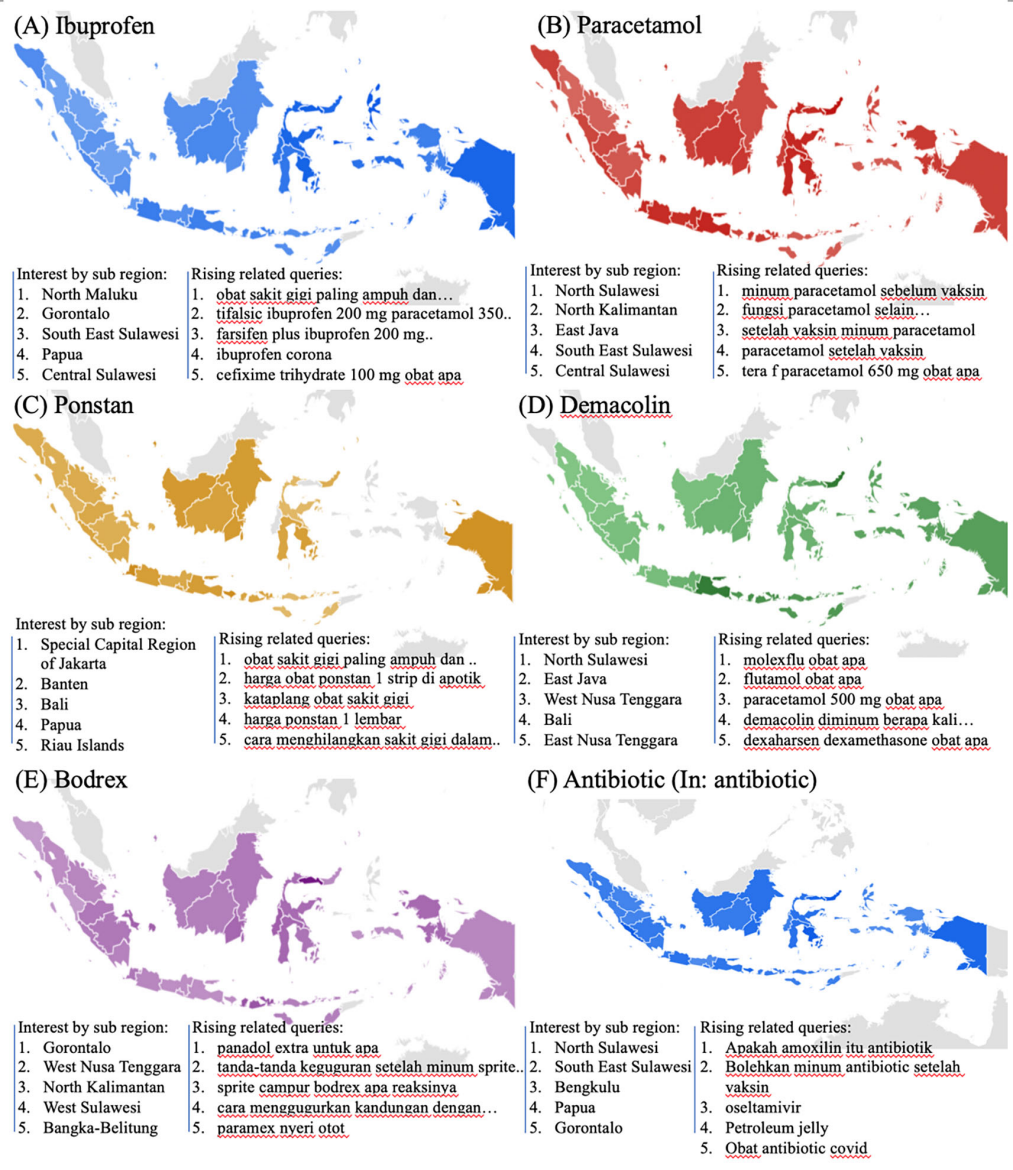
Google trend of drugs RSV curves-related search terms.

We examined the search interest related to drugs used during the COVID-19 pandemic. As seen in Figure 2B, there were six drugs that were commonly used during the COVID-19 pandemic (ibuprofen, paracetamol, ponstan, demacolin, bodrex, and antibiotic). Interestingly, those six drugs have shown to have a similar trend that increased during June 2021 and was down after August 2021. Figure 4A-F showed the top five sub-regions related to the drugs-search terms.

### Health managements

**Figure 5.**
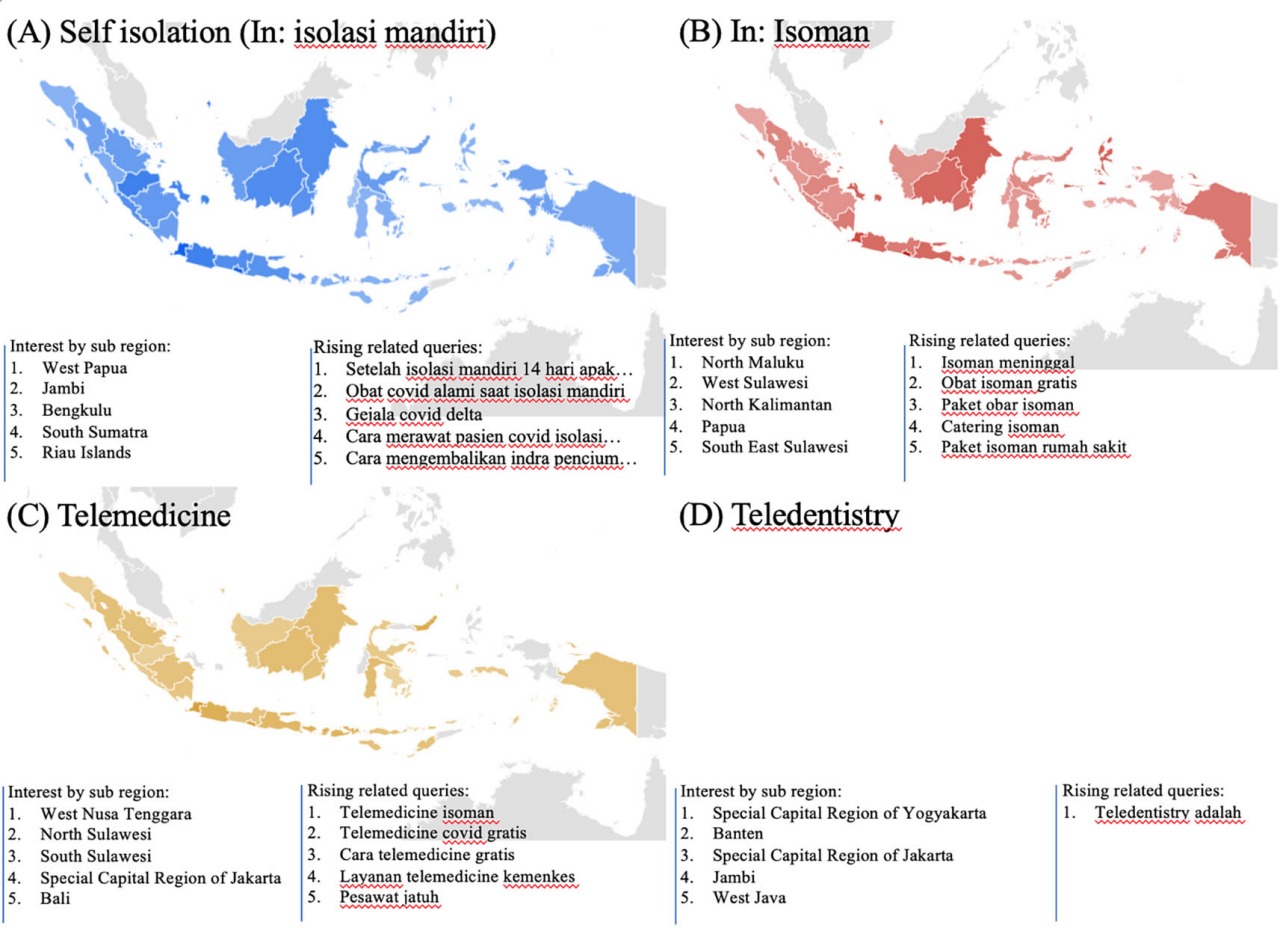
Google trend of health managements RSV curves-related search terms.

During the COVID-19 lockdown, RSVs curves revealed a rise in interest in terms associated to health management, including telemedicine, teledentistry, and self-isolation (In: isolasi mandiri or isoman) ( Figure 2C). Figure 4A–D listed the highest five searches by sub-region. The heat map in Figure 5D, however, could not be created due to little of data.

### Correlations among symptoms, drugs, health management-related RSVs, and COVID-19 Cases

As seen in Table 1, the daily confirmed COVID-19 cases were having a strong and significant positive correlation with COVID-19 using GT ( *P* < 0.01). The symptoms-related search terms (fever, sore throat, flu, toothache, and cough) were seen to have positive correlation with COVID-19 case (all *P* < 0.01). Similarly, drugs-related search term also had significant correlation with COVID-19 case (the *P*-values of ibuprofen, paracetamol, demacolin, and antibiotic were all *P* < 0.01, and ponstan was *P* < 0.05). For health management, the self-isolation, “isoman”, telemedicine but not teledentistry were significantly have correlation with COVID-19 case (all *P* < 0.01).

**Table 1.** Spearman’s correlation of symptoms, drugs, health managements, and COVID-19 data.

Fever	0.758 **	0.830 **	0.635 **	0.879 **	0.750 **	0.723 **	0.330 **	0.773 **	0.657 **	0.918 **	0.635 **	0.305 **	0.115	0.821 **	0.657 **	0.739 **
	Sore throat	0.850 **	0.599 **	0.852 **	0.589 **	0.555 **	0.308 **	0.716 **	0.608 **	0.773 **	0.460 **	0.277 **	0.085	0.693 **	0.564 **	0.528 **
		Flu	0.685 **	0.873 **	0.586 **	0.565 **	0.360 **	0.699 **	0.604 **	0.829 **	0.444 **	0.254 **	0.094	0.696 **	0.560 **	0.530 **
			Toothache	0.680 **	0.453 **	0.391 **	0.381 **	0.478 **	0.480 **	0.592 **	0.180 *	-0.001	0.086	0.447 **	0.279 **	0.306 **
				Cough	0.756 **	0.733 **	0.362 **	0.798 **	0.684 **	0.887 **	0.623 **	0.354 **	0.083	0.759 **	0.652 **	0.694 **
					Ibuprofen	0.823 **	0.248 **	0.735 **	0.569 **	0.744 **	0.755 **	0.501 **	0.043	0.665 **	0.673 **	0.736 **
						Paracetamol	0.264 **	0.718 **	0.617 **	0.718 **	0.849 **	0.560 **	0.090	0.653 **	0.772 **	0.774 **
							Ponstan	0.279 **	0.285 **	0.314 **	0.154 *	0.119	0.083	0.299 **	0.252 **	0.185 *
								Demacolin	0.565 **	0.792 **	0.659 **	0.456 **	0.050	0.731 **	0.711 **	0.711 **
									Bodrex	0.626 **	0.515 **	0.228 **	0.082	0.561 **	0.481 **	0.503 **
										Self-isolation	0.655 **	0.342 **	0.113	0.810 **	0.712 **	0.751 **
											“Isoman”	0.654 **	0.008	0.574 **	0.806 **	0.838 **
												Telemedicine	0.172 *	0.364 **	0.535 **	0.547 **
													Teledentistry	0.149 *	0.062	0.036
														Antibiotic	0.606 **	0.650 **
															COVID-19 GT	0.745 **
																COVID-19 WD

COVID-19 = Corona Virus Disease 2019, GT = Google Trends, WD = Our Worlds Data.

* *P* < 0.05.

** *P* < 0.01.

## Discussion

To the best of our knowledge, this is the first study investigating the symptoms trend, drugs, and health managements, during COVID-19 pandemic in Indonesia. We observed that there was a significant and positive correlation between the COVID-19 daily case using GT RSVs data and the COVID-19 case from Our World in Data. The COVID-19 daily case had a strong correlation with search terms related to symptoms (such as fever, sore throat, flu, toothache, and cough), drugs (such as ibuprofen, paracetamol, demacolin, bodrex, and antibiotic), and health management (such as self-isolation and telemedicine).

We found that symptoms-related terms such as fever and cough were significantly related to COVID-19 cases. In accordance with evidence that the health experts alerted the public to several primary characteristics of COVID-19, such as fever, persistent coughing, and an absence of taste and smell. ^25^ In contrast, toothache, the second higher symptom (see, Figure 2A), did not show any statistically significant result. However, with the highly infectious Delta variance, various oral symptoms might be emerging including dysgeusia, ageusia, a burning sensation in the mouth, a dry mouth, hyposmia, and severe halitosis ^26^^,^^27^ that may lead to toothache. Further investigations are warned.

Telemedicine is strongly correlated with COVID-19 based on this present study. The characteristic of the SARS-CoV-2 Delta variant implies that Delta may replicate more quickly and be more aggressive in the early stages of illness. ^28^ Compared to the Delta variation, the Omicron form is less likely to experience loss of or changes in smell, whereas wheezing of voice and sore throat are much more common. Acute symptom duration was greater for people with the Delta variant than for people with the Omicron variant. ^29^^,^^30^ Patients should be always encouraged to visit a doctor if they have worrying symptoms that are becoming worse. However, due to social distancing and lockdown, hence many patients were searching for terms related to telemedicine. In order to avoid infection transmission, a modification of inpatient treatment, such as using telemedicine, during the COVID-19 pandemic should be promoted.

Create awareness about the use of teledentistry should be emphasized to become a tool for patient’s oral health evaluation and a safe approach to start new treatments during COVID-19 pandemic, and for other diseases with similar pathway of transmission. However, from our findings, teledentistry has an insignificant correlation with COVID-19. These may be due to less knowledge of the patients or dentist to use teledentistry methods, that could lead to a low search level in internet. ^31^^,^^32^ Teledentistry is an effective method for screening the patient’s health condition previous to an *in person* dental consultation. ^16^^,^^17^ Using teledentistry for consultations before patients reach dental facilities in a pandemic situation should be incorporated for both, dentists and patients’ safety.

We acknowledge several limitations in this study. First, the timeframe was only focused on the specific time of April 2021 until September 2021. The application of the finding in another timeframe during the pandemic may be underestimated. However, that time frame was the important time to see the sever symptom and mortality rate due to COVID-19 in Indonesia. Second, the language-related search terms were used in the national language called Bahasa. As Indonesia has hundred local languages, those terms may be not adopted in this current study. However, since Bahasa become the only national language and is mostly considered the first language to share information on the internet, this current study was still rigorous. Lastly, GT did not measure symptoms directly but used the search term trends. The real symptom’s characteristics should be validated further.

## Conclusion

Using GT may be helpful to observe the current symptoms trends as well as its challenge tendencies as a surveillance tool for a continuing pandemic like COVID-19, particularly in the countries consisted of many islands and supported with proper internet accessed. GT should be considered and used as it has the potential to be a powerful digital epidemiology tool that can provide more insight into disease dynamics. Future improvements can be made, such as merging other digital data types such as Twitter and Facebook, in an attempt to increase the model’s capacity for prediction.

## Data Availability

COVID-19 case data available from: https://github.com/owid/covid-19-data/tree/master/public/data/ Google Trend data available from: at https://trends.google.com.tw/trends/?geo=TW. Search terms and other parameters are provided in the text. Data are available under the terms of the Creative Commons Attribution 4.0 International license (CC-BY 4.0).
