# Supplementary material for: Symptoms trend and challenges in dental practice during delta variance COVID-19 pandemic in Indonesia: Google Trends Analysis
Source: F1000Res. 2023 Aug 29;12:804. Originally published 2023 Jul 10. [Version 2] doi: 10.12688/f1000research.134366.2 (PMC10541538; doi:10.12688/f1000research.134366.2)
Supplement: Peer review history

# Reviewer report: Reviewer response for version 2

**DOI:** 10.5256/f1000research.154800.r201248

**Published:** 2023-09-29

**Authors:** Sri Susanty

**Affiliations:** 1 Nursing Professional Education Study Program, Faculty of Medicine, Halu Oleo University, Kendari, Indonesia

No further comments to make. Thank you for your hard work.

Is the work clearly and accurately presented and does it cite the current literature?

Partly

If applicable, is the statistical analysis and its interpretation appropriate?

Partly

Are all the source data underlying the results available to ensure full reproducibility?

Partly

Is the study design appropriate and is the work technically sound?

Partly

Are the conclusions drawn adequately supported by the results?

Partly

Are sufficient details of methods and analysis provided to allow replication by others?

Partly

Reviewer Expertise:

Gerontologies, healthy ageing, community health

I confirm that I have read this submission and believe that I have an appropriate level of expertise to confirm that it is of an acceptable scientific standard.

# Reviewer report: Reviewer response for version 2

**DOI:** 10.5256/f1000research.154800.r186201

**Published:** 2023-09-18

**Authors:** Merlya Balbeid

**Affiliations:** 1 Faculty of Dentistry, Universitas Brawijaya, Malang, Indonesia

Very interesting research, Google trends was used to discover Indonesian relative search volume (RSVs) covering the timeframe of the first outbreak of the COVID-19 pandemic in Indonesia. Bahasa was used to identify search items, but has limitations because Indonesia has a hundred local languages. It has potential to be a powerful digital epidemiology tool which can provide more insight into disease dynamics.

Is the work clearly and accurately presented and does it cite the current literature?

Yes

If applicable, is the statistical analysis and its interpretation appropriate?

Yes

Are all the source data underlying the results available to ensure full reproducibility?

Yes

Is the study design appropriate and is the work technically sound?

Yes

Are the conclusions drawn adequately supported by the results?

Yes

Are sufficient details of methods and analysis provided to allow replication by others?

Yes

Reviewer Expertise:

Public health

I confirm that I have read this submission and believe that I have an appropriate level of expertise to confirm that it is of an acceptable scientific standard.

## References

[1] : The correlation between the knowledge level related to practice protocols and dentists’ anxiety levels in practice during the COVID-19 pandemic. *Dental Journal (Majalah Kedokteran Gigi)* .2022;55(2) : 10.20473/j.djmkg.v55.i2.p99-104 99-104 10.20473/j.djmkg.v55.i2.p99-104

# Reviewer report: Reviewer response for version 1

**DOI:** 10.5256/f1000research.147417.r186204

**Published:** 2023-07-19

**Authors:** Sri Susanty

**Affiliations:** 1 Nursing Professional Education Study Program, Faculty of Medicine, Halu Oleo University, Kendari, Indonesia

The purpose of this manuscript is to help improve oral health status. However, I would like to point out some issues, as below:

1. What are the restrictions on dental care practice (dental issues) in relation to the “bahasa” search terms used by the authors? Please be descriptive (are they in the mouth, throat, teeth, or gums).
2. Please have a look at the list of medications that dentists frequently use based on the issue. You can utilize diclofenac, paracetamol, ibuprofen, and mefenamic acid if the issue is pain. This is significant because these medications cannot be used for other dental conditions.
3. In terms of the drug terminology used in your search terms, the drugs you list are still very general (there are symptomatic drugs to reduce pain symptoms, and there are causative bacteria). I suggest the authors group them.
4. What was the reason the authors used "demacolin and bodrex," which contain antihistamines while having a dental issue?
5. Why would the authors choose to use painkillers (ibuprofen, paracetamol, ponstan) or antibiotics for tooth infections? I suggest the authors write it clearly.
6. Why do the authors only take terminology from the patent name (brand) of a drug? And why were these brands chosen? Did the authors consider the price of branded drugs which are expensive and not necessarily used in dental health facilities?
7. Treatment of dental problems does not only use pharmacological therapy. However, Indonesia also uses traditional medicine and non-pharmacological therapy. Why don't the authors consider this?
8. Management of dental problems such as plaque does not always use oral medication, but also gargling with an antiseptic. Why did the authors not include mouthwash terminology? Why are the authors only considering oral medication? What about using albothyl and betadine? I know this is viral in Indonesia.
9. What about other dental problems such as canker sore? Generally, people use vitamin C before going to the doctor. Why did the authors not consider including the keyword vitamin C?
10. I failed to understand the use of antibiotics in the search item. What antibiotics did the authors use? Amoxicillin? Cotrimoxazole? Or penicillin? I suggest the authors consider this to avoid bias.

Is the work clearly and accurately presented and does it cite the current literature?

Partly

If applicable, is the statistical analysis and its interpretation appropriate?

Partly

Are all the source data underlying the results available to ensure full reproducibility?

Partly

Is the study design appropriate and is the work technically sound?

Partly

Are the conclusions drawn adequately supported by the results?

Partly

Are sufficient details of methods and analysis provided to allow replication by others?

Partly

Reviewer Expertise:

Gerontologies, healthy ageing, community health

I confirm that I have read this submission and believe that I have an appropriate level of expertise to confirm that it is of an acceptable scientific standard, however I have significant reservations, as outlined above.

# Response

**Published:** 2023-08-02

**Authors:** Faizul Hasan

**Affiliations:** Faculty of Nursing, Chulalongkorn University, Bangkok, Bangkok, Thailand

Reviewer 1 (Dr. Sri Susanty):

 The purpose of this manuscript is to help improve oral health status. However, I would like to point out some issues, as below:

**Response:** Thank you for your thorough review. We carefully considered your suggestions and comments and revised our manuscript accordingly. The following are our specific responses to each of the comments. We hope the revisions are satisfactory and that our responses adequately address the comments.

 What are the restrictions on dental care practice (dental issues) in relation to the “bahasa” search terms used by the authors? Please be descriptive (are they in the mouth, throat, teeth, or gums).

**Response:** The study primarily focuses on highlighting the symptoms, trends, and challenges in dental practice during the COVID-19 pandemic in Indonesia. The search term "sakit gigi" (translated to "toothache" in English) was used to emphasize major dental problems and related issues. However, regarding specific restrictions on dental care practice in relation to the "bahasa" search terms, the study does not provide explicit information on that matter. Instead, it aims to shed light on the impact of the pandemic on dental health and practices in Indonesia.

 Please have a look at the list of medications that dentists frequently use based on the issue. You can utilize diclofenac, paracetamol, ibuprofen, and mefenamic acid if the issue is pain. This is significant because these medications cannot be used for other dental conditions.

**Response:** We agree with you. Hence, in this study, we only focus on the frequent drug used to counter dental pain such as “ibuprofen” and “paracetamol”.

 In terms of the drug terminology used in your search terms, the drugs you list are still very general (there are symptomatic drugs to reduce pain symptoms, and there are causative bacteria). I suggest the authors group them.

**Response:** Thank you for your suggestion. In the first stage, we list several drugs and see the trend. However, some of the terms did not provide a good trend. Therefore, in the final results, we only include the term that showed a significant trend in association with COVID-19.

 What was the reason the authors used "demacolin and bodrex," which contain antihistamines while having a dental issue?

**Response:** We found that “demacolin” and “bodrex” have increased trends during the delta variance periods (see Figure 2B). As we know these two medications are generally and regularly used by Indonesian people if they feel unwell as the first self-medication before going to healthcare providers. Because the symptoms of COVID-19 include fever and flu, so the search terms for administration of these medications were high. In addition, some users considered taking medication (paracetamol, an ingredient of bodrex) before or after vaccination (see Figure 4B).

 Why would the authors choose to use painkillers (ibuprofen, paracetamol, ponstan) or antibiotics for tooth infections? I suggest the authors write it clearly.

**Response:** As mentioned in the response to question number 3, painkillers (ibuprofen, paracetamol, ponstan) or antibiotics provide increasing trends during the delta variance periods (Figure 2B). However, we are not sure whether this medication is used for tooth infection because it is out of our study aim. 

 Why do the authors only take terminology from the patent name (brand) of a drug? And why were these brands chosen? Did the authors consider the price of branded drugs which are expensive and not necessarily used in dental health facilities?

**Response:** As we explained in the previous response, firstly we include all related terms including generic and patent names of the drug, however, some of them did not provide good trends. As a note, Google trends provided data based on the users' search on Google machine but not the real symptom, so the final term included in this study actually the real-time search engine data. We already acknowledge this in the limitation section.

 Treatment of dental problems does not only use pharmacological therapy. However, Indonesia also uses traditional medicine and non-pharmacological therapy. Why don't the authors consider this?

**Response:** We also used the term for traditional medicine such as “jamu” or non-pharmacological therapy such as “pijat”. However, we did not find trends during the intended periods.

 Management of dental problems such as plaque does not always use oral medication, but also gargling with an antiseptic. Why did the authors not include mouthwash terminology? Why are the authors only considering oral medication? What about using albothyl and betadine? I know this is viral in Indonesia.

**Response:** We mainly focus on dental pain as it is considered the main problem. The other problem such as cleaning plaque was not our study aim. In addition, the term albothyl or betadine also did not provide a good trend during the study periods.

 What about other dental problems such as canker sore? Generally, people use vitamin C before going to the doctor. Why did the authors not consider including the keyword vitamin C?

**Response:** We did use the term “Vitamin C”, however, it did not show a significant trend.

 I failed to understand the use of antibiotics in the search item. What antibiotics did the authors use? Amoxicillin? Cotrimoxazole? Or penicillin? I suggest the authors consider this to avoid bias.

**Response:** The detail of the antibiotic was provided in Figure 4F. It included amoxicillin, oseltamivir, “obat antibiotic covid”.
